# Potential Benefits of Antiviral African Medicinal Plants in the Management of Viral Infections: Systematic Review

**DOI:** 10.3389/fphar.2021.682794

**Published:** 2021-12-24

**Authors:** Tamirat Bekele Beressa, Serawit Deyno, Andrew G. Mtewa, Namuli Aidah, Naasson Tuyiringire, Ben Lukubye, Anke Weisheit, Casim Umba Tolo, Patrick Engeu Ogwang

**Affiliations:** ^1^ Department of Pharmacy, College of Medicine and Health Sciences, Ambo University, Ambo, Ethiopia; ^2^ Pharm-Biotechnology and Traditional Medicine Center of Excellence, Mbarara University of Science and Technology, Mbarara, Uganda; ^3^ School of Pharmacy, Faculty of Medicine, Hawassa University, Hawassa, Ethiopia; ^4^ Chemistry Section, Department of Applied Studies, Institute of Technology, Malawi University of Science and Technology, Limbe, Malawi; ^5^ School of Nursing and Midwifery, College of Medicine and Health Sciences, University of Rwanda, Butare, Rwanda; ^6^ Department of Biology, Faculty of Science, Mbarara University of Science and Technology, Mbarara, Uganda

**Keywords:** SARS-CoV-2 (2019-nCoV), medicinal plants, viral infections, Africa, herbal mecidine

## Abstract

**Background:** Viruses cause various human diseases, some of which become pandemic outbreaks. This study synthesized evidence on antiviral medicinal plants in Africa which could potentially be further studied for viral infections including Coronavirus disease 2019 (COVID-19) treatment.

**Methods:** PUBMED, CINAHIL, Scopus, Google Scholar, and Google databases were searched through keywords; antiviral, plant, herb, and Africa were combined using “AND” and “OR”. *In-vitro* studies, *in-vivo* studies, or clinical trials on botanical medicine used for the treatment of viruses in Africa were included.

**Results:** Thirty-six studies were included in the evidence synthesis. Three hundred and twenty-eight plants were screened for antiviral activities of which 127 showed noteworthy activities against 25 viral species. These, were Poliovirus (42 plants), HSV (34 plants), Coxsackievirus (16 plants), Rhinovirus (14plants), Influenza (12 plants), Astrovirus (11 plants), SARS-CoV-2 (10 plants), HIV (10 plants), Echovirus (8 plants), Parvovirus (6 plants), Semiliki forest virus (5 plants), Measles virus (5 plants), Hepatitis virus (3 plants), Canine distemper virus (3 plants), Zika virus (2 plants), Vesicular stomatitis virus T2 (2 plants). Feline herpesvirus (FHV-1), Enterovirus, Dengue virus, Ebola virus, Chikungunya virus, Yellow fever virus, Respiratory syncytial virus, Rift Valley fever virus, Human cytomegalovirus each showed sensitivities to one plant.

**Conclusion:** The current study provided a list of African medicinal plants which demonstrated antiviral activities and could potentially be candidates for COVID-19 treatment. However, all studies were preliminary and *in vitro* screening. Further *in vivo studies* are required for plant-based management of viral diseases.

## Background

Viruses cause various human diseases of which several such as Ebola, HIV/AIDS, and Hepatitis B are hard to treat. Many pandemic outbreaks in world history were caused by a viral infection. The Spanish flu pandemic of 1918, the deadliest in history, infected an estimated 500 million people worldwide; which is about one-third of the planet’s population, and killed an estimated 20 million to 50 million people (1). In recent years, pandemics have arisen and have also been contained using various approaches. For example, Ebola virus outbreak between 2013 and 2016 with 11323 deaths (Trilla et al., 2008), Coronavirus (Severe Acute Respiratory Syndrome (SARS) with deaths of 229 (World Health Organization, 2003), Middle East respiratory syndrome (MERS) as of May 31, 2015, which had 483 (40%) mortality (Zumla et al., 2015) are some of the recorded global pandemics. Since December 2019 the world is suffering from Coronavirus disease 2019 (COVID-19) with more than 197 million people infected and more than 4, 219, 861 deaths as of August 4, 2021 (World Health Organization, 2020).

The use of natural medicinal agents dates back to human prehistory where plants formed the basis of traditional medicine (TM) systems. Traditional medicine refers to health practices, approaches, knowledge, and beliefs incorporating plant, animal, and mineral-based medicines, spiritual therapies, manual techniques, and exercises which are applied singularly or in combination to treat or to diagnose and prevent illnesses or maintain well-being (World Health Assembly, 2003). Traditional medicine has a high influence on the African health system with an estimated 80% of the population depending on TM practice for primary health care purposes (World Health Organization, 2005). The availability and affordability of the TM aligned with inherited knowledge of the practice in local communities might have contributed to their wide use (Fennell et al., 2004).

Several herbal medicines have been used to treat viral infections traditionally for a long time. Some studies have reported the inhibitory effect of medicinal plant extracts against several viruses. Some of these studies were conducted on HIV, herpes simplex virus, hepatitis B virus, and poliovirus. For example, ethnobotanical studies in Africa described the treatment of viral hepatitis with traditional medicine in Africa (Vlietinck et al., 1995; Sindambiwe et al., 1999; Cos et al., 2002a; Amenu, 2007; Abera, 2014; Traore et al., 2018). Furthermore, plants have been reported to have antiviral potential against conventional medicine-resistant strains of viruses (Serkedjieva, 2003). Nine traditional Chinese botanicals were optimized to treat the symptoms of SARS during its outbreak (Zhang et al., 2004). In another study, small molecules from natural compounds have been screened and confirmed to inhibit important proteins in SARS or MERS coronavirus (Zhang et al., 2020). Despite having lots of endemic knowledge and practice on African herbal medicine, there is a paucity of scientific evidence on their efficacy and safety. This study aimed to summarize the evidence on antiviral medicinal plants in Africa which could potentially be further studied for COVID-19 treatment.

## Methods

### Study Design

This review was conducted using database searches and followed statements for Reporting Systematic Reviews and Meta-Analyses (Liberati et al., 2009).

### Search Strategy

Data were collected from MEDLINE/PUBMED, CINAHIL, Google Scholar, and Scopus databases. No language limitations were applied to reduce selection bias and Google was used to translate articles published in other languages than English. The search strategy used the following terms with appropriate Boolean operators; (“virus diseases” OR (“virus” AND “diseases”) OR “virus diseases” OR (“viral” AND “infection”) OR “viral infection”) OR (“poliovirus” OR “poliovirus” OR HSV OR (“simplexvirus” OR “simplexvirus” OR (“herpes” AND “simplex” AND “virus”) OR “herpes simplex virus”) OR (“enterovirus” OR “enterovirus” OR “coxsackievirus” OR (“influenza, human” OR (“influenza” AND “human”) OR “human influenza” OR “influenza”) OR (astro AND (“viruses” OR “viruses” OR “virus”)) OR (“parvovirus” OR “parvovirus”) OR (“rhinovirus” OR “rhinovirus”) OR (“enterovirus b, human” OR “human enterovirus b” OR “echovirus”) OR (“hiv"OR “hiv”) OR (“hiv”OR “hiv” OR (“human” AND “immunodeficiency” AND “virus”) OR “human immunodeficiency virus”) OR (semiliki AND (“forests”OR “forests” OR “forest”) AND (“viruses”OR “viruses” OR “virus”)) OR (“measles virus”OR (“measles” AND “virus”) OR “measles virus”) OR (“hepatitis viruses”OR (“hepatitis” AND “viruses”) OR “hepatitis viruses” OR (“hepatitis” AND “virus”) OR “hepatitis virus”) OR (“zika virus”OR (“zika” AND “virus”) OR “zika virus”) OR ((“vesicular stomatitis indiana virus”OR (“vesicular” AND “stomatitis” AND “indiana” AND “virus”) OR “vesicular stomatitis indiana virus” OR (“vesicular” AND “stomatitis” AND “virus”) OR “vesicular stomatitis virus”) AND T2) OR (“coronavirus disease 2019” OR “COVID-2019″) AND “herbal medicine” OR “traditional medicine” OR “oriental medicine” OR “Chinese medicine” OR “African medicine” OR “herbal formula” OR herb AND”) AND (“ AND (“africa"OR “africa”) AND “OR” AND ((“african continental ancestry group”OR (“african” AND “continental” AND “ancestry” AND “group”) OR “african continental ancestry group” OR “african”) AND countries).

### Study Selection

We included original research articles and unpublished dissertations from their inception to 2020. The unpublished dissertations were obtained from university website (http://etd.aau.edu.et, http://erepository.uonbi.ac.ke). EndNote reference manager was used to remove the duplications of references before screening. Either *in vitro* studies or *in vivo* studies or clinical trials of herbal medicine on African medicinal plants were included. Studies were eligible for inclusion if they were conducted to determine antiviral activities using available scientific methods and conducted on medicinal plants in Africa. Studies conducted on medicinal plants outside of Africa were excluded from the study. Review articles and ethnobotanical studies were also excluded. Eligibility assessment was conducted by TB and SD independently and disagreement between authors was resolved by discussion.

## Results

**FIGURE 1:**
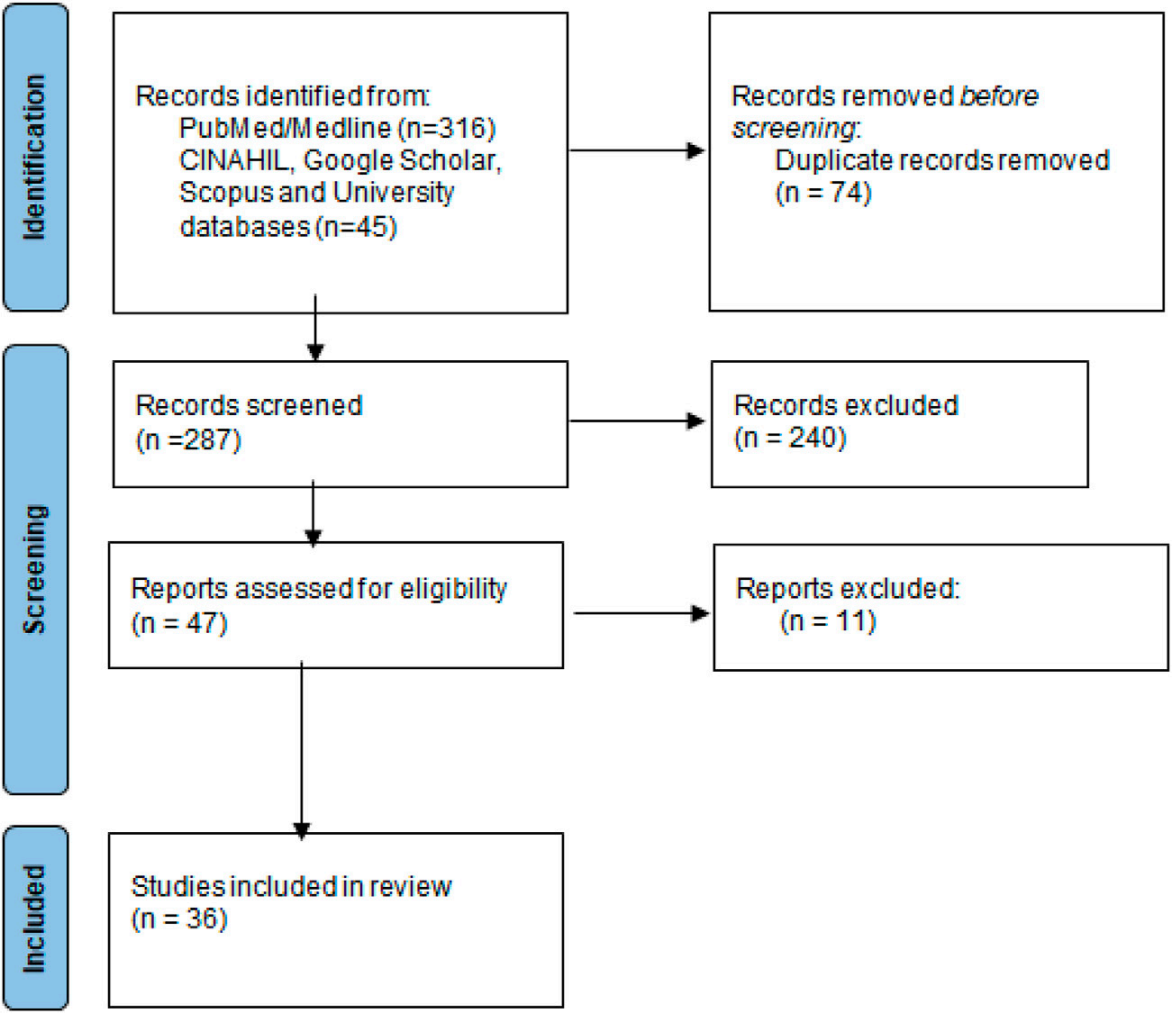
Flow diagram of included studies. Legend: The PRIMSA diagram details our search and selection process applied during the review.

In this study 316 publications were retrieved of which 36 (Ferrea et al., 1993; Beuscher et al., 1994; Vlietinck et al., 1995; Nakano et al., 1997; Kitamura et al., 1998; Hussein et al., 1999; Kudi and Myint, 1999; Sindambiwe et al., 1999; Anani et al., 2000; Yoosook et al., 2000; Cos et al., 2002b; Chiang et al., 2003; Wang et al., 2004; Bessong et al., 2005; Gebre-Mariam et al., 2006; Tolo et al., 2006; Kambizi et al., 2007; Maregesi et al., 2008; Duraipandiyan and Ignacimuthu, 2009; Gyuris et al., 2009; Ojo et al., 2009; Selvarani, 2009; Sunday et al., 2010; Astani et al., 2011; Nwodo et al., 2011; Sultana, 2011; Ndhlala et al., 2013; Ogbole et al., 2013; Kwena, 2014; David et al., 2017; Clain et al., 2018; Mehrbod et al., 2018; Nasr-Eldin et al., 2018; Ogbole et al., 2018; Cambaza, 2020; Gyebi et al., 2021) were included in the qualitative synthesis, Figure 1.

Three hundred and twenty-eight plants were screened for antiviral activities of which 127 tested showed activities against 25 viral species; Among these were Poliovirus (42 plants), HSV (34 plants), Coxsackievirus (16 plants), Rhinovirus (14plants), Influenza (12 plants), Astrovirus (11 plants), SARS-CoV-2 (10 plants), HIV (10 plants), Echovirus (8 plants), Parvovirus (6 plants, Semiliki forest virus (5 plants), Measles virus (5 plants), Hepatitis virus (3 plants), Canine distemper virus (3 plants), Zika virus (2 plants), Vesicular stomatitis virus T2 (2 plants). Feline herpes virus (FHV-1), Enterovirus, Dengue virus, Ebola virus, Chikungunya virus, Yellow fever virus, Respiratory syncytial virus, Rift Valley fever virus, Human cytomegalovirus each showed sensitivities to one plant (Tables 1–4). Isolated compounds were also identified and their activities outlined, namely alkaloids (combretine and betonicine) from Combretum micrantum (Ferrea et al., 1993), Aloin from *Aloe ferox* (Kambizi et al., 2007), a polysaccharide from *Aspalathus. Linearis* (Nakano et al., 1997), Asiaticoside from *Centella asiatica* (Yoosook et al., 2000), Catechin from *S. frutescens* (Bessong et al., 2005)*.*

**TABLE 1:** Antiviral activity of African medicinal plants against HIV virus.

Species, Family	Parts used	Extracting solvent	Activity	References
*Aspalathus linearis* (Burm.f.) R.Dahlgren (Fabaceae)	L	Alkaline water	Active against HIV with (EC_50_ = 38.9 μg/ml)	Nakano et al. (1997)
*Croton megalobotrys* Müll.Arg. (Euphorbiaceae)	R	Methanol	Activates latent HIV-1 provirus in J-lat cells at 0.5 μg/ml = 1.3 ± 0.2%	Tietjen et al. (2016)
*Euphorbia hirta* L. (Euphorbiaceae)	AP	Methanol	Active againist HIV-1, with (IC_50_ = 5 6 0.5 μg/ml)	Gyuris et al. (2009)
*Hypericum revolutum Vahl* (Hypericaceae)	L	Ethanol	Active against HIV-1 with EC_50_ > 131.13 μg/ml and CC_50_ > 131.13 μg/ml	Cos et al. (2002b)
*Microglossa pyrifolia* (Lam.) Kuntze (Asteraceae)	S	Ethanol	Active againist HIV-1 with EC_50_ > 140.1 μg/ml, and CC_50_ = 140.1 μg/ml)	Cos et al. (2002b)
*Sutherlandia frutescens* (L.) R.Br. (Fabaceae)	L	Methanol	Active against HIV RNA-dependent DNA polymerase (RDDP) IC_50_ = 2000 μg/ml, RNase H IC_50_ >100 μg/ml	Bessong et al. (2005)
		Methanol	Acts on HIV RNA-dependent DNA polymerase (RDDP) with IC_50_ = 2000 μg/ml, and RNase H IC_50_ >100 μg/ml	
*Terminalia sericea Burch. ex DC.* (Rutaceae)	L	Methanol	Inhibits HIV-1 RDDPby (98%); HIV-1, and RNase inhibition by 99.3%	Bessong et al. (2005)
*Triumfetta rhomboidea* Jacq. (Malvaceae)	L	Ethanol	Active against HIV-1 with EC_50_ ≥0.03, and CC_50_ = 0.03 μg/ml)	Cos et al. (2002a)
*Triumfetta rhomboidea* (Tiliaceae)	L	Ethanol	Active against HIV-1 with EC_50_ > 0.03 and CC_50_ = 0.03	Cos et al. (2002b)

AP, areal part; L, leaf; S, stem; R, root; CC_50_, The 50% cytotoxic concentration; DNA, deoxyribonucleic acid; EC_50_, Half maximal effective concentration; HIV-1, human immunodeficiency virus type 1; IC_5,_ Half-maximal inhibitory concentration; RNA, ribonucleic acid.

**TABLE 2:** Antiviral activity of African medicinal plants against Influenza virus.

Species, Family	Parts used	Extracting solvent	Activity	References
*Acokanthera schimperi* (A.DC.) Schweinf. (Apocynaceae)	L	Hexane	Inhibited parainfluenza virus production by 50% at 1–10 dilution factor	Bagla et al. (2012)
*Aspalathus linearis* (Burm.f.) R.Dahlgren (Fabaceae)	L	Alkaline	Inhibited influenza A and Bvirus production by 50%	Rahmasaria et al. (2017)
*Adansonta digitata* L.(Bombacaceae)	L	Methanol	Active againist Influenza A (H_3_N_2_) virus human isolate with MIC of 0.72 μg/ml	Selvarani, (2009)
	L	DMSO	Active againist Influenza A (H_3_N_2_) virus human isolate with MIC of 0.12 μg/ml annd RSV with MIC = 16.2 μg/ml	Selvarani, (2009)
	AP			Clain et al. (2018)
*Carissa spinarum* L. (Apocynaceae)	L	hexane	Inhibited parainfluenza virus by 25% at a 1 to 10 dilution	Bagla et al. (2012)
*Rotheca myricoides* var. discolor (Klotzsch) Verdc. (Lamiaceae)	L	Methanol	Active against influenza A virus with EC_50_ = 110.4 μg/ml and CC_50_ = 221 ± 34.9 μg/ml)	Mehrbod et al. (2018)
*Helichrysum armenium* DC. (Asteraceae)	L	Water and ethanol	Inhibited parainfluenza virus with MIC of 4 μg/ml	Bagla et al. (2012)
*Helichrysum melanacme* DC. (Asteraceae)	L	Ethanol	Inhibited influenza A virus production with IC_50_ of 10 μg/ml	Rahmasaria et al. (2017)
*Pavetta ternifolia* Hiern. Rubiaceae)	L	Methanol, 30 and 100% ethanol, Acetone	Active against influenza A virus. For acetone extract with EC_50_ = 82.3 μg/ml and CC_50_ = 165 ± 25.2 μg/ml	Mehrbod et al. (2018)
			For ethanol (30%) CC_50_ = 77 ± 24.8 μg/ml, EC_50_ = 19.2 μg/ml for ethanol (100%) CC_50_ = 7 ± 5.8 μg/ml andEC_50_ = 3.4 μg/ml SI = 2; For methanol CC_50_ = 15 ± 9.3 μg/ml and EC_50_ = 3.6 μg/ml SI = 4	
*Pelargonium sidoides* DC. (Geraniaceae)	Not specified	EPs 7630	Inhibited the replication of influenza A H_1_N_1_ and H_3_N_2_ at the concentration of 100 μg/ml	
*Pterocarpus angolensis* DC. (Fabaceae)	SB	Methanol	Active against influenza A virus with CC_50_ = 227 ± 13.6 and EC_50_ = 113.3	Mehrbod et al. (2018)
	B, F, L			
*Rapanea melanophloeos* (L.) Mez (Primulaceae)	L	Water, methanol,ethanol, aceton	Inhibited inlfuenza A virus with EC_50_ of 113 μg/ml	Mehrbod et al. (2018), More et al. (2021)
*Sterculia setigera* Delile (Malvaceae)	L	Hexane	Active against influenza A virus (EC_50_ = 4.7 μg/ml)	Lu et al. (2005), Duraipandiyan and Ignacimuthu, (2009)

AP, areal part; B, bark; L, leaf; SB, stem bark, R, root; RB, root bark; WP, whole plant; F, fruit; DMSO, dimethyl sulfoxide; CC_50_, the 50% cytotoxic concentration; EC_50_, half maximal effective concentration; MIC, minimum inhibitory concentration.

**TABLE 3:** Antiviral activity of African medicinal plants against Herpes simplex virus.

Species, Family	Parts used	Extracting solvent	Activity	References
*Adansonta digitata* L. (Bombacaceae)	RB, L	Methanol	Active against HSV with MIC 65.5 μg/ml	Anani et al. (2000)
*Aloe ferox* Mill. (Xanthorrhoeaceae)	L	Water	Active against HSV-1 with MIC = 63 μg/ml	Kambizi et al. (2007)
*Anogeissus leiocarpa* (DC.) Guill. and Perr. (Combretaceae)	L	Ethanol	Showed 50% inhibition of HSV1 and Equine HSV	Kudi and Myint, (1999)
*Bauhinia thonningii* Schum. (Leguminosae)	L	Ethanol	Showed total inhibition of HSV 1, Equine HSV, and 75% inhibition of Bovine HSV	Kudi and Myint, (1999)
*Bidens pilosa* L. (Compositae)	WP	Hot water	Inhibited HSV-1 with ED_50_ of 655.4 μg/ml and for HSV-2 with ED_50_ of 960 μg/ml	Chiang et al. (2003)
*Centella asiatica* (L.) Urb. (Apiaceae)	AP	Water	Inhibited HSV-1 with Ec_50_ of 362.40 μg/ml	Yoosook et al. (2000)
*Carissa spinarum* L. (Apocynaceae)	R, B	Water	Active against HSV with CC_50_ of 480 μg/ml	Tolo et al. (2006), Kwena (2014)
*Chironia krebsii* Griseb. (Capparaceae)	R	DCM	Active against HSV in the EC range of 6.25–12.5 μg/ml, SI = 2	Beuscher et al. (1994)
*Rotheca myricoides* (Hochst.) Steane & Mabb. (Lamiaceae)	L, R	Ethanol	Active against HSV with RF 10^3^	Vlietinck et al. (1995), Sindambiwe et al. (1999)
*Clutia abyssinica* Jaub. and Spach (Peraceae)	L	Ethanol	Active against HSV with RF of 10^3^	Vlietinck et al. (1995)
*Combretum micranthum* G.Don (Combretaceae)	L	Ethanol	Active against HSV-1 with EC_50_ of 2 μg/ml	Ferrea et al. (1993)
			Active against HSV-2 with EC_50_ of 4 μg/ml	
*Crassocephalum macropappus (Sch.Bip. ex A.Rich.) S.Moore* (Compositae)	L	Ethanol	Active against HSV with RF of 10^3^	Vlietinck et al. (1995)
*Detarium senegalense* J.F.Gmel. (Leguminosae)	L	Ethanol	Inhibit Astrovirus HSV 1, Equine HSV at effective concentration of 2 mg/ml	Kudi and Myint (1999)
*Dichrostachys cinerea* (L.) Wight & Arn. (Fabaceae)	L	Ethanol	Inhibit HSV 1, Equine HSV, at effective concentration of 1 mg/ml	Kudi and Myint (1999)
*Dryopteris inaequalis* (Schltdl.) Kuntze (Dryopteriaceae)	WP	Ethanol	Active against herpes with 10^3^ viral titer factor reduction	Vlietinck et al. (1995)
*Erigeron aegyptiacus* L. (Compositae)	L	Methanol	Active against HSV with MIC of 500 μg/ml	Anani et al. (2000)
*Eriosema montanum* Baker f. (Fabaceae)	L	Ethanol	Active against HSV with RF = 10^4^	Cos et al. (2002a)
*Euphorbia hirta* L. (Euphorbiaceae)	WP		Active against HSV with RF 10^3^	Vlietinck et al. (1995)
*Helichrysum foetidum* (L.) Cass. (Compositae)	WP	Ethanol	Virucidal against HSV 1 with MVC >1/20	Sindambiwe et al. (1999)
*Neonotonia wightii* (Wight & Arn.) J.A.Lackey (Fabaceae)	L, S	Ethanol	Active against HSV with RF 10^3^ virus	Vlietinck et al. (1995)
*Guiera senegalensis* J.F.Gmel. (Combretaceae)	L	Ethanol	Inhibits HSV1 and Equine HSV	Kudi and Myint, (1999)
*Guizotia Scabra* (Vis.) Chiov. (Asteraceae)	L	Ethanol	Active against the HSV virus with RF of 10^3^	Cos et al. (2002a)
*Houttuynia cordata* Thunb. (Saururaceae)		Hot water	Inhibited replication of HSV. The Ec_50_ of HSV-1 was822.4 μg/ml and HSV-2 was 362.5 μg/ml.	Chiang et al. (2003)
*Ipomoea bonariensis* Hook. (Convolvulaceae)	AP	Ethanol	Showed true antiviral activity against HSV1 with RF of 10 and MVC of 1/100	Sindambiwe et al. (1999)
*Jasminum fluminense* Vell. (Oleaceae)	S	ethanol	Active against HSV from cc50-200 μg/ml, SI = 2	Beuscher et al. (1994)
*Lannea humilis* (Oliv.) Engl. (Anacardiaceae)	B	Ethanol	Inhibit HSV 1and Equine HSV with EC of 1 mg/ml	Kudi and Myint, (1999)
*Leonotis nepetaefolia* var. africana (P.Beauv.) J.K.Morton (Lamiaceae)	F	Ethanol	Active against HSV with RF of 10^2^	Vlietinck et al. (1995)
*Maesa lanceolata* Forssk. (Myrsinaceae)	L	Ethanol	Virucidal activity against HSV1 with MVC 1/400	Sindambiwe et al. (1999)
*Moringa oleifera* Lam. (Moringaceae)	L	Water	Active against HSV-1 with %inhibition of 43.2 and HSV-2 with % inhibition of 21.4	Nasr-Eldin et al. (2018)
*Markhamia lutea* (Benth.) K.Schum. (Bignoniaceae)	R, L	Ethanol	Active against HSV with RF 0f 10^3^	Vlietinck et al. (1995)
*Mitragyna inermis* (Willd.) Kuntze (Rubiaceae)	L	Methanol	Active against HSV with EC from 50–100 μg/ml; SI = 2	Beuscher et al. (1994)
*Palisota hirsute* (Thunb.) K.Schum. (Commelinaceae)	L	Methanol	Active against HSV (MIC = 62.5 μg/ml)	Anani et al. (2000)
*Rubus rigidus* Sm. (Rosaceae)	L, R	Ethanol	Antiviral activity against HSV (RF of 10^4^)	Vlietinck et al. (1995)
*Securidaca longepedunculata Fresen.* (Polygalaceae)	R	Methanol	Active against HSV with EC from 12.5–25 μg/ml SI = 2	Beuscher et al. (1994)
*Sterculia setigera* Delile (Sterculiaceae)	L	Ethanol	Showed total Inhibition of HSV 1 and Equine HSV with of 1 mg/ml	Kudi and Myint, (1999)

AP, areal part; B, Bark; L, leaf; SB, stem bark; R, root; RB, root bark; WP, whole plant; HSV, herpes simplex virus; DCM, dichloromethane; SI, Selective index; EC50, half maximal effective concentration; MVC, minimal virucidal concentration; RF, reduction factor of viral titre.

**TABLE 4:** Antiviral activity of African medicinal plants against poliovirus, astrovirus, coxsackievirus, Rift Valley fever virus, zika virus, measle, echovirus, yellow fiver virus, parvovirus, chikungunya virus, cytomegalovirus, CDV.

Species, Family	Parts used	Extracting solvent	Activity	References
*Acacia sieberiana* DC. (Fabaceae)	L, R, B	Ethanol	Active against coxsackievirus with RF of 10^5^	Vlietinck et al. (1995)
*Adansonia digitata* L. (Malvaceae)	L	DMSO	Inhibited Rift Valley fever virus with DPPH EC50 Of 4.64 μg/ml and ABTS EC50 5.04 μg/ml	More et al. (2021)
*Aphloia theiformis* (Vahl) Benn. (Aphloiaceae)	AP	solvent free	Inhibit zika virus entry into host cells atIC50 = 100 μg and CC50 = 3000 μg/ml; SI = 30	Clain et al. (2018)
*Aframomum melegueta* K.Schum. (Zingiberaceae)	SB	Ethanol	Active against Measles Virus with MIC = 125 μg/mLandYellow Fiver Virus with MIC of250 μg/mL.	Ojo et al. (2009)
*Ageratum conyzoides* L. (Compositae)	L	Methanol	Active against Ecovirus with CC50 of 155.33 μg/ml	Ogbole et al. (2018)
*Anacardium occidentale* L. (Anacardiaceae)	B, L		Showed total inhibition of Poliovirus, Astrovirus, Bovine parvovirus, Canine parvovirus	Kudi and Myint, (1999)
*Anogeissus leiocarpa* (DC.) Baill. (Combretaceae)	L	Ethanol	Showed total inhibition of poliovirus andastrovirus	Kudi and Myint, (1999)
*Artemisia afra* Jacq. (Asteraceae)	L	DMSO	Inhibited Rift Valley fever virus with DPPH EC50 Of 20.41 μg/ml and ABTS EC50 16.39 μg/ml	More et al. (2021)
*Baccharoides lasiopus (O.Hoffm.) H.Rob.* (Compositae)	L, S	Ethanol	Active against coxsackie virus with of RF 10^2^	Vlietinck et al. (1995)
*Badula insularis* A.DC. (Primulaceae)	L	DCM	Active against rhinovirus type 2 with EC range from 2.5–5 μg/ml SI = 2	Beuscher et al. (1994)
*Bauhinia thonningii* Schum. (Leguminosae)	L	Ethanol	Showed total inhibition of Poliovirus andAstrovirus; 75% inhibition of Bovine parvovirus, Canine parvovirus	Kudi and Myint, (1999)
*Bryophyllum pinnatum* (Lam.) Oken (Crassulaceae)	L	Methanol	Inhibited echovirus with CC_50_ of 125.47 μg/ml; IC_50_ againist E7 strainwas3.13 μg/ml; and IC_50_ against E19 strain was 2.03 μg/ml	Ogbole et al. (2018)
*Cajanus cajan* (L.) Millsp. (Fabaceae)	L,S,R	Water, ethanol	Active against coxsackie virus with RF of 10^3^	Vlietinck et al. (1995), Nwodo et al. (2011)
*Capparis tomentosa* Lam. (Capparaceae)	L, S	notspecified	Active against coxsackie virus with RF of 10^4.5^	Vlietinck et al. (1995)
*Carissa edulis* L. (Carissa edulis)	L	Hexane	Active against FHV-1 and CDV with EC50 of 73.17and 12.37 respectively	More et al. (2021)
*Rotheca myricoides (Hochst.) Steane & Mabb.* (Verbenaceae)	L, R	Ethanol	Active against coxsackie virus, with RF 10^2^	Vlietinck et al. (1995), Sindambiwe et al. (1999)
*Solanecio mannii* (Hook.f.) C.Jeffrey (Compositae)	L	Ethanol	Active against Coxsackie with RF of 10^3^	Vlietinck et al. (1995)
*Crassula globularioides subsp.* argyrophylla (Diels ex Schönland and Baker f.) Toelken (Crassulaceae)	AP	DCM	Active against Rhinovirus with EC range from 6.25–25 μg/ml, SI = 4; Poliovirus with EC range from 12.5–25 μg/ml, SI = 2	Beuscher et al. (1994)
		Methanol	Active against Rhinovirus EC range from 6.25–25 μg/ml μg/ml SI = 2; Poliovirus EC range from 50–100 μg/ml SI = 2	Beuscher et al. (1994)
		Ethanol	Active against rhinovirus with EC from 6.25–25 μg/ml μg/ml, SI = 2	Beuscher et al. (1994)
*Crinum jagus* (J.Thomps.) Dandy (Amaryllidaceae)	B	Methanol	Active against Echovirus with CC_50_ of 9.88 μg/ml	Ogbole et al. (2018)
*Crotalaria mesopontica* Taub. (Fabaceae)	L, S	Ethanol	polio virus with RF of 10^3^	Vlietinck et al. (1995)
*Cussonia spicata* Thunb. (Araliaceae)	WP	Methanol	Active against Coxsackievirus with CC_50_ of 117 ± 11.5 μg/ml and EC50 of14.6 μg/ml; SI = 8	Sultana, (2011)
		Ethanol	CC50 = 39 ± 12.6 μg/ml EC50 = 4.8 μg/ml, SI = 8	
		Acetone	Active against Coxsackie virus: Acetone CC_50_ = 108 ± 2.4 μg/ml m, EC50 = 13.5 μg/ml SI = 8	
*Detarium senegalense* J.F.Gmel. (Leguminosae)	L	Ethanol	Inhibit Poliovirus, Astrovirus, Bovine parvovirus, Canine parvovirus with an effective concentration of 2 mg/ml	Kudi and Myint, (1999)
*Dichrostachys cinerea* (L.) Wight & Arn. (Fabaceae)	L	Ethanol	Inhibit Poliovirus Astrovirus, Bovine parvovirus, Canine parvovirus with an effective concentration of 1 mg/ml	Kudi and Myint, (1999)
*Dracaena elliptica* Thunb. and Dalm. (Asparagaceae)	R	Ethanol	Active against coxsackie with RF 10^3^	Vlietinck et al. (1995)
	F	Ethanol	Active against polio virus and coxsackie with 10^4^ and 10^3^ viral titer reduction factor respectively	
*Dryopteris inaequalis* (Schltdl.) Kuntze (Dryopteriaceae)	WP	Ethanol	Active against poliovirus with 10^3^ viral titer factor reduction	Vlietinck et al. (1995)
*Ekebergia capensis* Sparrm. (Meliaceae)	L	DCM	Active agaist CDV with EC50 of 30.93 respectively	More et al. (2021)
*Elaeodendron croceum* (Thunb.) DC. (Celastraceae)	L	DMSO	Inhibited Rift Valley fever virus with DPPH EC50 of 6 μg/ml and ABTS EC50 4.12 μg/ml	More et al. (2021)
*Elaeodendron transvaalense* (Burtt Davy) R.H.Archer (Celastraceae)	L	DMSO	Inhibited Rift Valley fever virus with DPPH EC50 of 11.64 μg/ml and ABTS EC50 15 μg/ml	More et al. (2021)
*Elephantorrhiza elephantina* (Burch.) Skeels (Fabaceae)	L	DMSO	Inhibited Rift Valley fever virus with DPPH EC50 of 6.54 μg/ml and ABTS EC50 7.4 μg/ml	More et al. (2021)
*Eriosema montanum* Baker f. (Fabaceae)	L	Ethanol	Active against Coxsackie virus with RF of 10^3^, measles with RF 10^2^, Poliovirus with RF of 10^3^, SF with RF of 10^4^ andVSV with RF of 10^2^	Cos et al. (2002a)
*Erythrina abyssinica*DC.(Fabaceae)	S, R, L	Ethanol	Active against polio, semiliki forest and measles virus with RF of 10^4^	Vlietinck et al. (1995)
*Euclea natalensis* A.DC. (Ebenaceae)	L	DMSO	Inhibited Rift Valley fever virus with DPPH EC_50_ of 5.3 μg/ml and ABTS EC_50_ of 5.00 μg/ml	More et al. (2021)
*Helichrysum abietifolium* Humbert (Asteraceae)	L	DMSO	Inhibited Rift Valley fever virus with DPPH EC_50_ of 8.25 μg/ml and ABTS EC_50_ 11.4 μg/ml	More et al. (2021)
*Euphorbia grantii* Oliv. (Euphorbiaceae)	L, S	Ethanol	Active against poliovirus and Coxsackie virus with RF of 10^5^	Vlietinck et al. (1995)
*Euphorbia hirta* L. (Euphorbiaceae)	WP	Not specified	Active against poliovirus with RF of 10^5^ and against Coxsackie virus with RF of 10^3^	Vlietinck et al. (1995)
*Guiera senegalensis* J.F.Gmel*.* (Combretaceae)	L	Ethanol	Inhibits poliovirus	Kudi and Myint, (1999)
*Guizotia Scabra* (Vis.) Chiov. (Asteraceae)	L	Ethanol	Active against the Coxsackie and Poliovirus with RF of 10^3^	Cos et al. (2002a)
*Heteromorpha arborescens* (Spreng.) Cham. and Schltdl. (Apiaceae)	RB	Methanol	Active against Poliovirus with EC from 10–25 μg/ml SI = 2.5	Beuscher et al. (1994)
	RB	Ethanol	Active against Poliovirus with EC from 12.5–50 μg/ml SI = 4	Beuscher et al. (1994)
*Hibiscus sabdariffa* L. (Malvaceae)	L	Ethanol	Active against measles virus at with EC from 10–15 mg/ml	Sunday et al. (2010)
*Helichrysum cymosum* (L.) D.Don (Compositae)	WP	Ethanol	Showed virucidal activity against Semiliki forest virus A7 with RF of 10^3^	Sindambiwe et al. (1999)
*Holarrhena pubescens* Wall. ex G.Don (Appocynaceae)	SB	DCM	Active against rhinovirus with EC range from 10–25 μg/ml SI = 2.5	Beuscher et al. (1994)
		EtOH	Active against rhinovirus with EC range from 50–25 μg/ml SI = 2.5	Beuscher et al. (1994)
*Ipomoea asarifolia* (Desr.) Roem. and Schult*.* (Convolvulaceae)	L	Methanol	Showed potent antiviral activity against Echo virus With CC_50_ of 84.21 μg/ml	Ogbole et al. (2018)
*Ipomoea bonariensis* Hook. (Convolvulaceae)	AP	Ethanol	Showed virucidal effect against vesicular stomatitis virus T2 (VSV T2) with RF of 103	Sindambiwe et al. (1999)
*Jasminum fluminense* Vell. (Appearance)	S	DCM	Active against Poliovirus with EC range of 100–200 μg/ml, SI = 2	Beuscher et al. (1994)
		Methanol	Active against Poliovirus with EC range of 100–400 μg/ml, SI = 4	Beuscher et al. (1994)
		Ethanol	Active against Poliovirus with EC range of 50–1200 μg/ml, SI = 24	Beuscher et al. (1994)
		Methanol	Active against rhinovirus with EC range of 50–100 μg/ml, SI = 2	Beuscher et al. (1994)
*Khaya senegalensis* (Desv.) A.Juss. (Meliaceae)	B	Ethanol	Inhibit Poliovirus, Astrovirus with EC of 2 mg/ml	Kudi and Myint, (1999)
*Labourdonnaisia calophylloides* Bojer (Sapotaceae)	L	DCM	Active against Poliovirus with EC range from 5–200 μg/ml, SI = 40	Beuscher et al. (1994)
		Ethanol	Active against Poliovirus with EC range from 12.5 to 25 μg/ml, SI = 2	Beuscher et al. (1994)
		Ethanol	Active against rhinovirus with EC range from 25–50 SI = 2	Beuscher et al. (1994)
*Laggera brevipes* Oliv. and Hiern (Fabaceae)	L, S, F	Ethanol	Active against poliovirus and Coxseckie virus with RF of 10^3^ and 10^4^ respectively	Vlietinck et al. (1995)
*Lannea humilis* (Oliv.) Engl. (Anacardiaceae)	B	Ethanol	Inhibit Poliovirus and Astrovirus with EC of 1 mg/ml	Kudi and Myint, (1999)
*Leonotis nepetaefolia* (L.) R.Br. (Lamiaceae)	F	Ethanol	Active against coxsakievirus withRF of 10^2^	Vlietinck et al. (1995)
*Lippia multiflora* Moldenke (Verbenaceae)	L	Ethanol	Active against Echovirus with CC_50_ of 112.07 μg/ml	Ogbole et al. (2018)
*Maesa lanceolata* Forssk. (Myrsinaceae)	L	Ethanol	Virucidal activity against Measles vurus with MVC of 1/800	Sindambiwe et al. (1999)
*Macaranga barteri* Müll.Arg. (Euphorbiaceae)	L	Methanol	Active against serotypes of enterovirus (E7, E13 and E19) with CC_50_ () of 0.27 μg/ml	Ogbole et al. (2018)
*Macaranga kilimandscharica* Pax (Euphorbaceae)	L	Ethanol	Active against Poliovirus with RF of 10^3^	Vlietinck et al. (1995)
*Mitragyna inermis* (Willd.) Kuntze (Rubiaceae)	L	DCM	Active against Poliovirus with EC from 12.5–25 μg/ml, SI = 2	Beuscher et al. (1994)
		Methanol	Active against Poliovirus with EC from 25–200 μg/ml, SI = 8	Beuscher et al. (1994)
		DCM	Active against rhinovirus With EC from 12.5–25 μg/ml	Beuscher et al. (1994)
*Mondia whitei* (Hook.f.) Skeels (Periplocaceae)	L	Methanol	Active against echovirus with CC50 of 132.50 μg/ml	Ogbole et al. (2018)
*Myonima violacea* (Lam.) Verdc. (Rubiaceae)	L	DCM	Active against Poliovirus with EC from 6.3–50 μg/ml, SI = 8	Beuscher et al. (1994)
		Ethanol	Active against Poliovirus with EC from 25–50 μg/ml, SI = 2	Beuscher et al. (1994)
		DCM	Active against rhinovirus with EC from 20–50 μg/ml, SI = 2	Beuscher et al. (1994)
		Ethanol	Active against rhinovirus EC of 50–60 μg/ml SI = 2	Beuscher et al. (1994)
*Pavetta ternifolia* Hiern. (Rubiace)	L	Ethanol	Showed virucidal activities against enveloped viruses with MVC>1/20 and slightly active extracellularly against VSV with MVC = 1/20	Sindambiwe et al. (1999)
*Plantago palmate* Lam. (Plantaginaceae)	L	Ethanol	Active against coxsakie (RF 10^3^, polio (RF 10^1.5)^ virus	Vlietinck et al. (1995)
*Plumbago zeylanica* L. (Plumbaginaceae)	L	Hexane	Active against CDV with EC50 of 11.73	Bagla et al. (2012)
*Polygala stenopetala* Klotzsch (Polygalaceae)	AP	DCM	Active against Poliovirus with EC range from 100–400 μg/ml, SI_=_ 4	Beuscher et al. (1994)
		Ethanol	Active against Poliovirus with EC range from 100–200 μg/ml, SI = 2	Beuscher et al. (1994)
*Polygala virgate* Polygala virgate (Polygalaceae)	AP	DCM	Active against Poliovirus with EC range from 12.5–100 μg/ml, SI = 8	Beuscher et al. (1994)
		Methanol	Active against Poliovirus with EC range from 25–100 μg/ml, SI = 4	Beuscher et al. (1994)
		Ethanol	Active against Poliovirus with EC from 50–400 μg/ml, SI = 4	Beuscher et al. (1994)
		DCM	Active against rhinovirus with EC range from 12.5–25 μg/ml, SI = 2	Beuscher et al. (1994)
		Methanol	Active against rhihinovirus with EC range from 25–100 μg/ml, SI = 4	Beuscher et al. (1994)
		ethanol	Active against rhinovirus with EC range from 50–200 μg/ml, SI = 4	Beuscher et al. (1994)
*Polygonum pulchrum* (Blume) Soják (Polygalaceae)	R	Ethanol	Active against Coxsackievirus with RF 10^3^	Vlietinck et al. (1995)
*Prunus africana* (Hook.f.) Kalkman (Rosaceae)	SB	Water	Active against HCMV with EC_50_ of 80 μg/ml	Tolo et al. (2007)
*Psiloxylon mauritianum* (Bouton ex Hook.f.) Baill. (Myrtaceae)	AP	Solvent-free microwave	Active against Zika and Dengue virus with CC_50_ of 1044 g/ml (Vero cells); CC_50_ of 657 g/ml (A549 cells); CC_50_ of 353 g/ml (keratinocytes); CC_50_ of 820 g/ml (fibroblast); SI = 53.5	Clain et al. (2018)
*Pterocarpus angolensis* DC. (Fabaceae)	SB	Methanol	Active against Poliovirus with EC range from 50–100 μg/ml, SI = 2	Beuscher et al. (1994)
		Ethanol	Active against Poliovirus with EC range from 50–100 μg/ml, SI = 2	Beuscher et al. (1994)
		Ethanol	Active against rhinovirus with EC range from 12.5–25 μg/ml, SI = 2	Beuscher et al. (1994)
*Searsia pyroides* (Burch.) Moffett (Anacardiaceae)	L, R	Ethanol	Antiviral activity against Semiliki forest and Coxsackievirus with RF of 10^4^	Vlietinck et al. (1995)
*Rubus rigidus* Sm. (Rosaceae)	L, R	Ethanol	Antiviral activity against Semiliki forest virus Coxsackievirus with RF of 10^4^	Vlietinck et al. (1995)
*Securidaca longepedunculata* Oliver (Polygalaceae)	R	DCM	Active against poliovirus with EC from 5–10 μg/ml, SI = 2	Beuscher et al. (1994)
		Methanol	Active against poliovirus with EC from 5–10 μg/ml, SI = 2	
*Senna siamea* (Lam.) H.S.Irwin & Barneby (Fabaceae)	B	Methanol	Active against poliovirus with a ratio of CC_50_ to IC_50_ = 0.0019	Ogbole et al. (2013)
*Senna singueana* (Delile) Lock (Leguminosae)	L	Not specified	Inhibit Poliovirus, Astrovirus, Bovine parvovirus	Kudi and Myint, (1999)
*Sideroxylon puberulum* A.DC. (Sapotaceae)	L	DCM	Active against poliovirus with EC range from 10–50 μg/ml SI, = 5	Beuscher et al. (1994)
*Solanum incanum* L. (Solanaceae)	R, F	Ethanol	Antiviral activity against Coxsackievirus with RF of 10^4^	Vlietinck et al. (1995)
*Spondias dulcis* Parkinson (Anacardiaceae)	B, L	Methanol	Active against Echovirus with CC_50_ of 53.33 μg/ml	Ogbole et al. (2018)
*Steganotaenia araliacea* Hochst. (Apiaceae)	R	Methanol	Active against rhinovirus with EC range from 5–10 μg/ml, SI = 2	Beuscher et al. (1994)
*Sterculia setigera* Delile (Sterculiaceae)	L	Ethanol	Inhibit Poliovirus, Astrovirus, Bovine parvovirus, Canine parvovirus with a total inhibition at EC of 1 mg/ml	Kudi and Myint, (1999)
*Sutherlandia frutescens* (L.) R.Br. (Fabaceae)	L	DMSO	Inhibited Rift Valley fever virus with DPPH EC_50_ of 32.2 μg/ml and ABTS EC_50_ of 42.3 μg/ml	More et al. (2021)
*Tabernaemontana ventricosa* Hochst. ex A.DC. (Apocynaceae)	L	Methanol	Antiviral activity against poliovirus with CC_50_ of 0.1 ± 0.07 μg/ml and EC_50_ of 0.05 μg/ml; SI = 2	Mehrbod et al. (2018)
*Terminalia ivorensis*A.Chev. (Combretaceae)	B	Methanol	Active against Echovirus with CC_50_ of 12.14 μg/ml	Ogbole et al. (2018)
*Tetracera alnifolia* Willd. (Dilieniaceae)	L	Methanol	Active against echovirus CC_50_ of 147.8 μg/ml	Ogbole et al. (2018)
*Tabernaemontana ventricosa* Hochst. ex A.DC. (Apocynaceae)	L	Methanol	Active against poliovirus with CC_50_ of 0.1 ± 0.07 μg/ml; EC50 of 0.05 μg/ml; SI = 2	Mehrbod et al. (2018)
*Terminalia ivorensis* A.Chev. (Combretaceae)	B	Methanol	Active against Echovirus withCC_50_ = 12.14 μg/ml	Ogbole et al. (2018)
*Tetracera alnifolia* Willd. (Dilieniaceae)	L	Methanol	Active against Echovirus with CC_50_ of 147.8 μg/ml	Ogbole et al. (2018)
*Voacanga Africana* Stapf ex Scott-Elliot (Apocynaceae)	RB	Water	Active against Chikungunya viral disease	Ndhlala et al. (2013)
*Vernoniastrum aemulans* (Vatke) H.Rob. (Compositae)	L	Ethanol	Active against Poliovirus with RF of 10^4^	Vlietinck et al. (1995)
*Vernonia amygdalina* Del. (Compositae)	F	Ethanol	Active against poliovirus with RF of 10^3^	
*Vitellaria paradoxa* C.F.Gaertn. (Sapotaceae)	B	Ethanol	50% inhibition of Poliovirus and Astrovirus	Kudi and Myint (1999), Ogbole et al. (2013)
*Xanthocercis madagascariensis* Baill. (Fabaciae)	L	DCM	Active against poliovirus with EC from 25–50 μg/ml; SI = 2	Beuscher et al. (1994)
		Methanol	Active against poliovirus with EC from 25–100 μg/ml; SI = 4	
		Ethanol	Active against poliovirus with EC from 500–1000 μg/ml; SI = 2	
		Methanol	Active against rhinovirus with EC from 60 to 80 μg/ml; SI = 1.6	
*Zanha Africana* (Radlk.) Exell (Sapindaceae)	RB	DCM	Active against poliovirus with EC from 12.5–25, SI = 2	
*Zephyranthes candida* (Lindl.) Herb. (Amaryllidaceae)	WP	Methanol	Active against poliovirus with the ratio of CC_50_ to IC_50_ 0.21 μg/ml	Ogbole et al. (2013)
*Ziziphus mucronata*Willd. (Rhamnaceae)	L	Ethanol	75% inhibition Poliovirus and Astrovirus with EC of 2 mg/ml	Kudi and Myint, (1999)

AP, areal part; B, bark; L, leaf; SB, stem bark; R, root; RB, root bark; WP, whole plant; HSV, herpes simplex virus; HCMV, human cytomegalovirus; RSV, respiratory syncytial virus; DPPH, 2,2-diphenyl-1-picrylhydrazayl; ABTS, 2,2 azino-bis(3-ethaylbenzothiazoline-6-sulfonic acid); DCM, dichloromethane; DMSO, dimethyl sulfoxide; SI, selective index; CC_50_, the 50% cytotoxic concentration; EC_50_, Half maximal effective concentration; IC_50_, the half-maximal inhibitory concentration; F, reduction factor of viral titre; CDV, canine distemper virus.

## Discussion

This study summarized the antiviral activities of African medicinal plants. Forty two African medicinal plants showed noteworthy activities against poliovirus and twenty four against HSV.

### Medicinal Plants Used for Severe Acute Respiratory Syndrome

Recently, 10 African medicinal plants from Morocco showed noteworthy activities against SARS-CoV-2 (58). However, there is no currently available published study on Africa medicinal plants demonstrating clinical effectiveness. In contrast, China has developed several Chinese herbal medicines (CHM) and produced numerous clinical studies and publications. There is a daring absence of published studies on herbal medicine use in Africa in comparison to the actual magnitude of its practice. Many Africans are using one or another type of African traditional medicine either for prevention or treatment of COVID-19.

For example, Madagascar produced an herbal drink from *Artemisia annua* called COVID Organics which was even exported abroad (Cambaza, 2020). The anecdotal use of this product resulted in exaggerated claims of their efficacies that are not evidence-based. This calls for the urgent need for further research on this as well as all other herbal formulations on their efficacy through randomized controlled trials and identify their active ingredients, develop proven formulations and dosing protocols, and define pharmacokinetics, toxicology, and safety to enable drug development. Derivatives from the herb *Artemisia annua* have been used for the treatment of fevers, malaria, and respiratory tract infections. The WHO has offered to support the design of a study to assess the efficacy, safety, and dosage formulation of herbal formulations that may be useful against COVID-19 (Muhammad, 2020). The WHO is currently helping the validation of some traditional medicine through clinical trials for the treatment of COVID-19 (Tih, 2020).

Studies on TM use for COVID-19 produced many publications of which four were systematic reviews and meta-analyses entirely based on CHM (Liu et al., 2006; Fan et al., 2020; Liu et al., 2020; Xiong et al., 2020) and other systematic reviews and meta-analyses were not CHM (Ang et al., 2020). Traditional medicine is being used to control coronavirus alone or in a combination with western medicine. A recent systematic review and meta-analysis of randomized controlled trials included seven randomized controlled trials and compared combined therapy of herbal medicine with Western medicine and western medicine alone (Ang et al., 2020). This demonstrated the potential role of herbal medicine in treating and/or managing COVID-19 (Ang et al., 2020). The other study which included 12 randomized controlled trials and one quasi-RCT with A total of 640 SARS-CoV-2 patients and 12 Chinese herbs did not indicate a significant difference in Chinese herbs combined with Western medicines versus Western medicines alone (Liu et al., 2006). Yet hundreds of Chinese traditional medicines had been widely used for the treatment of SARS and currently, it’s being used for SARS-CoV-2 (Shahrajabian et al., 2020). A recent review conducted by Attah et al. (2021) summarized 17 African medicinal plants studied against Covid-19 with viral protein targeted. The medicinal plants listed targeted SARS-Cov-2 3CLpro and ACE2.

An *in silico* screening was conducted on 62 alkaloids and 100 terpenoids from African medicinal plants against coronavirus 3-chymotrypsin-like protease (3CL ^pro^), a highly defined hit-list of seven compounds. Furthermore, four nontoxic, druggable plant-derived alkaloids and terpenoids that bind to the receptor-binding site and catalytic dyad of SARS-CoV-2 3CL^pro^ were identified. More than half of the selected top 20 alkaloids and terpenoids had a binding affinity for the 3CL^pro^ of the SARS-coronaviruses that surpassed reference inhibitors. The 6-oxoisoiguesterin from *Bisnorterpenes* had the highest binding affinity to the 3CL^pro^ of SARS-CoV-2 while 20-epi-isoiguesterinol from *Bisnorterpenes,* isoiguesterin from *Bisnorterpenes,* 20-epibryonolic acid from *Cogniauxia podolaena* was the top docked compounds to 3CL^pro^ of SARS-CoV and MERS-CoV. The study revealed that natural agents from the alkaloids and terpenoids class of compounds are capable of inhibiting the 3CL^pro^ with a high inhibitory pattern to both SARS-CoV-2 and SARS-CoV (Gyebi et al., 2021). Moreover, 67 compounds from Moroccan aromatic and medicinal plants were tested by molecular docking, of which 11 molecules showed good interaction with the studied enzyme [(Coronavirus (2019-nCoV) main protease] and three molecules Crocin, Digitoxigenin, b-Eudesmol had shown better interaction Coronavirus (2019-nCoV) main protease) (Aanouz et al., 2021). Crocin, a compound from *Crocus Sativus*, inhibited the replication of HSV (Soleymani et al., 2018). Digitoxigenin is a compound from *Nerium oleander* and studied for its antiviral and anticancer activity (Boff et al., 2019). Β-Eudesmol was extracted from *Lauris nobilis* has significant antiviral activity (Astani et al., 2011).

### Medicinal Plants for Ebola Virus

Medicinal plants target viruses through various mechanisms*. Garcinia kola*’*s* A 13 components showed activity against Ebola virus probably by binding with membrane proteins, metalloproteases, and Ser/Thr Kinase through the three most featured targets; cannabinoid receptors, cyclin-dependent kinases, and matrix metalloproteinase. The components could also target cathepsin, collagenase, and another matrix metalloproteinase (King, 2000; Homsy et al., 2004; David et al., 2017). Baicalin from (*Scutellariae Radix*), a natural product from the plant, acts on chemokine receptors and inhibits the entry of HIV (Kitamura et al., 1998; Li et al., 2000; Wang et al., 2004). The N-butanol fraction of *Bredelia micrantha* showed reverse transcriptase inhibition activity. Terpenes showed an inhibitory effect against the protease enzyme (Hussein et al., 1999; Huang and Chen, 2002; Tolo et al., 2006; Yu et al., 2006).

### Medicinal Plants for HIV

There are different targets for HIV drug developments. One is the viral envelope which plays a major role in infecting a cell by interacting with CD4 and chemokine receptors CCR5 and CXCR4. CV-N and Baicalin is a natural product from a plant source that acts on chemokine receptors and inhibits the entry of HIV (Kitamura et al., 1998; Li et al., 2000; Wang et al., 2004). The reverse transcriptase enzyme is also a target for drug development. The study comparing organic solvent and an aqueous fraction of various medicinal plants, and the *n*-butanol fraction of Bredelia micrantha showed anti-reverse transcriptase activities. Phytochemicals such as terpenes revealed inhibitory effects against protease enzyme; an important enzyme for proteolytic processing of polyprotein precursor into essential proteins for the assembly of virus particles (Hussein et al., 1999; Huang and Chen, 2002; Yu et al., 2006).

Croton megalobotrys is a plant species which showed the latent HIV-1 reversal activity. Crude extractas of the plant was comparable with known LRA prostatin which induced HIV-1 in J-lat cells. From the fraction of the crude extract, two novel phorbol esters (Namusha1 and 2) were identified. The previous study also showed that multiple phorbol esters had anti-HIV-1 activities (El-Mekkawy et al., 2000) and function as LRAs (Tietjen et al., 2018).

### Medicinal Plants for Hepatitis Virus

Medicinal plants have been widely used to treat the hepatitis virus. Out of five plants examined for anti-Hepatitis B virus, three exhibited anti-hepatitis B *in vitro* with a CC50 value of more than 100 μg/ml. These were aqueous extracts from *Carissa edulis* (Apocynaceae), *Prunus africana* Kalkman (Rosaceae) and the methanol extract from *Acacia mellifera* Benth (Fabaceae). Extracts of *C. edulis* exhibited the highest activity; an over 12.15% inhibition rate relative to the negative control. *P. africana* and *A. mellifera* extract demonstrated 5% inhibition and 2.15% inhibition respectively, relative to controls. Further confirmation of the activity of these plants using the quantitative real-time PCR technique showed the aqueous extract of *C. edulis* and the methanol extract of *A. mellifera* exhibited sustained activity over a range of plant extract concentrations from 31.25 μg/ml to 125 μg/ml. The evaluation of the EC_50_ the two plant extracts exhibiting notable anti–HBV activity using this technique yielded; *C. edulis’* EC_50_ was 331.6 μg/ml while that of *A. mellifera* was 295.0 μg/ml (Kwena, 2014).

### African Medicinal Plants for Influenza Virus

Influenza virus infection remains a major health problem for animals and humans. Medicinal plants are becoming increasingly popular and included in primary health care in different parts of the world. A study conducted on methanol, ethanol, acetone, hot and cold aqueous extract of five plants (*Pittosporum viridiflorum*, *Cussonia spicata*, *Rapanea melanophloeos*, *Tabernaemontana ventricosa*, *Clerodendrum glabrum*) against influenza A virus exhibited antiviral effect. Most effective result were obtained from *Rapanea melanophloeos* methanol leaf extract (EC_50_ = 113.3 μg/ml) and *Pittosporum viridiflorum* methanol, 100 and 30% ethanol and acetone leaf extracts (EC_50_ values = 3.6, 3.4, 19.2, 82.3 μg/ml, respectively) (Mehrbod et al., 2018). Ethiopian medicinal plants like *Acokanthera schimperi*, *Euclea schimperi*, leaf extracts of *Inula confertiflora* prevent influenza A virus replication and those of *Melilotus elegans* were active against influenza A virus (Gebre-Mariam et al., 2006) (Table 2).

### Medicinal Plants for Herpes Simplex Virus

In sub-Saharan Africa, high prevalence rates between 60 and 80% in young adults have been recorded in population-based studies. It is usually managed by antiviral drugs such as a nucleoside analog acyclovir. However, resistance to ACV has been reported mainly among immunocompromised patients (Morfin and Thouvenot, 2003). Medicinal plants have been considered as an alternative for the development of a new drug to overcome the resistance to the modern drug. The study was conducted on an aqueous extract from the root bark of *Carissa edulis* (Apocynaceae) has shown significant anti-HSV activity *in vitro* and *in vivo* (Omino and Kokwaro, 1993). The extract significantly inhibited the formation of plaques in Vero E6 cells infected with 100 PFU of the wild-type strains of HSV by 100% at 50 μg/ml *in vitro* with minimal cell cytotoxicity (Tolo et al., 2006). The extracts from four plants; *Lannea schweinfurthii*, *Combretum adenogonium*, *Ficus sycomorus*, and *Terminalia mollis* showed strong antiviral activity against Herpes Simplex Virus type 1. Out of 42 Egyptian medicinal plants, *Ephedra alata* and *Moringa peregrina* are found to have antiviral activity against HSV. Also, the results revealed that *Capparis sinaica*, *Tamarix nilotica*, and *Cyperus rotundus* are found to have a virucidal effect against HSV(Soltan and Zaki, 2009).

The current study is only a preliminary study where some studies reported naively. As all studies *in vitro* possible dose range, duration of action and *in vivo* pharmacodynamics properties cannot be established.

In conclusion, African medicinal plants pose significant antiviral activities and could potentially be candidates for viral disease treatment and/or management. It is imperative therefore that research on currently available African medicinal plants be highly recommended. Outcomes from such studies would potentially lead to breakthrough discoveries for the management and/or treatment of COVID-19 and various other viral infections upon appropriate optimization.

## Data Availability

The original contributions presented in the study are included in the article/Supplementary Material, further inquiries can be directed to the corresponding author.

## Abbreviations

CC_50_, 50% cytotoxic concentration; COVID-19, coronavirus diseases 2019; EC_50_, half maximal effective concentration; HIV, human immune deficiency virus; HSV, herpes simplex virus; MERS-CoV, middle east respiratory syndrome coronavirus; PRISMA, preferred reporting items for systematic reviews and meta-analysis; SARS-CoV, severe acute respiratory syndrome coronavirus; SI, selective index; CHM, chinese herbal medicine.
